# The Construction of a Lattice Image and Dislocation Analysis in High-Resolution Characterizations Based on Diffraction Extinctions

**DOI:** 10.3390/ma17030555

**Published:** 2024-01-24

**Authors:** Kun Ni, Hanyu Wang, Qianying Guo, Zumin Wang, Wenxi Liu, Yuan Huang

**Affiliations:** School of Materials Science and Engineering, Tianjin University, Tianjin 300354, China

**Keywords:** HRTEM, dislocation, Fourier transform, Burgers vector

## Abstract

This paper introduces a method for high-resolution lattice image reconstruction and dislocation analysis based on diffraction extinction. The approach primarily involves locating extinction spots in the Fourier transform spectrum (reciprocal space) and constructing corresponding diffraction wave functions. By the coherent combination of diffraction and transmission waves, the lattice image of the extinction planes is reconstructed. This lattice image is then used for dislocation localization, enabling the observation and analysis of crystal planes that exhibit electron diffraction extinction effects and atomic jump arrangements during high-resolution transmission electron microscopy (HRTEM) characterization. Furthermore, due to the method’s effectiveness in localizing dislocations, it offers a unique advantage when analyzing high-resolution images with relatively poor quality. The feasibility of this method is theoretically demonstrated in this paper. Additionally, the method was successfully applied to observed edge dislocations, such as $1/6[21\overset{-}{1}]$, $1/6[\overset{-}{2}1\overset{-}{1}]$, and $1/2[0\overset{-}{1}1]$, which are not easily observable in conventional HRTEM characterization processes, in electro-deposited Cu thin films. The Burgers vectors were determined. Moreover, this paper also attempted to observe screw dislocations that are challenging to observe in high-resolution transmission electron microscopy. By shifting a pair of diffraction extinction spots and superimposing the reconstructed images before and after the shift, screw dislocations with a Burgers vector of $1/2[01\overset{-}{1}]$ were successfully observed in electro-deposited Cu thin films.

## 1. Introduction

The high-resolution transmission electron microscope (HRTEM) is a widely used technique for characterizing the microstructures of materials [1,2,3,4,5,6,7]. It is an imaging technique that is based on the phase contrast principle [8] and comprises two fundamental processes: the incident electron beam scatters after passing through a thin crystal specimen and undergoes Fourier transformation in the back focal plane of the objective lens, forming a diffraction pattern that carries crystal structure information. Subsequently, interference between the transmitted beam and multiple diffracted beams occurs, leading to Fourier inverse transformation to reconstruct high-resolution images of the crystal structure in the image plane of the objective. Clearly, the formation of high-resolution images involves the two mathematical processes of Fourier transformation and inverse transformation [9].

Using high-resolution transmission electron microscopy, one can obtain one- and two-dimensional lattice images and structural images, with two-dimensional lattice images and structural images being directly applicable to the observation of crystal defects such as dislocations within the crystal. Due to the abundance of information in actual lattice and structural images, it is often challenging to visually discern the distribution and features of certain crystal planes. Simultaneously, in cases where the dislocation analysis of specific regions of lattice images is required, there may be difficulties due to poor image clarity. In such cases, Fourier transformation can be applied to the regions of interest in the lattice images, specific diffraction spots of particular crystal planes can be selected, noise can be filtered, and Fourier inverse transformation can be performed to reconstruct the fringe images of specific crystal planes, facilitating the localization and analysis of dislocations on those planes. However, due to the limitations of transmission electron microscopy principles, the information of certain crystal planes may be hidden, even if they satisfy Bragg diffraction conditions, causing diffraction extinction phenomena. Diffraction extinction is a very common phenomenon during TEM observations. It is characterized by the absence of an extinction spot at a location where it should theoretically exist. For different crystal structures, diffraction extinction shows different patterns. This is manifested during specific microscopic observations as the inability to find corresponding diffraction spots for these crystal planes in the diffraction pattern, making it challenging to directly analyze the arrangement patterns of these crystal planes. For example, in face-centered cubic (FCC) crystal structures, crystal planes with indices like {011} and {012} follow the “odd-even mixed” extinction rule, leading to the absence of corresponding spots in the electron diffraction pattern and Fourier transform spectrum. Thus, these spots cannot be directly selected for Fourier inverse transformation to reconstruct the corresponding fringe images, making it difficult to observe their dislocation distribution. Nevertheless, it is crucial to observe these types of crystal planes, as comprehensive analyses of full dislocations such as $a/2\langle 110\rangle$ and partial dislocations such as $a/6\langle 112\rangle$ and $a/3\langle 112\rangle$ are essential for a more detailed understanding of phenomena such as dislocation reactions [10,11], stacking faults, twinning mechanisms [12,13], and the strengthening mechanisms of certain metals [14,15].

**Figure 1:**
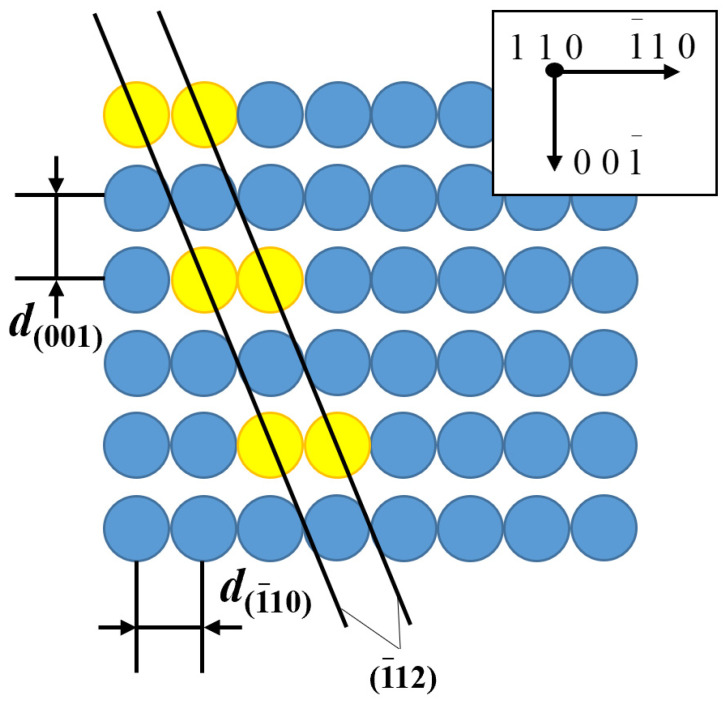
Schematic diagram of face-centered cubic crystal plane $\left(\overset{-}{1}12\right)$ and its atom arrangement without diffraction spots but with extinction.

Furthermore, the atomic arrangement on crystal planes that exhibit diffraction extinction often features a jumping arrangement under certain crystal zone axes, such as the distribution of the $\left(\overset{-}{1}12\right)$ plane in an FCC crystal under the [110] zone axis, as illustrated in Figure 1. This particular atomic arrangement makes direct observation of these crystal planes in lattice images challenging. In such cases, it becomes even more necessary to rely on fringe images for localization. However, in conventional approaches, due to the disappearance of corresponding diffraction spots, it becomes impossible to reconstruct fringe images, rendering localization operations unfeasible. Additionally, there is an ongoing challenge in the current high-resolution lattice imaging techniques concerning the observation of edge dislocations, which is relatively easier to achieve, while the observation of screw dislocations remains more challenging.

This research introduces a novel method for characterizing metal dislocations, termed the “High-Resolution Lattice Image Reconstruction and Dislocation Analysis Based on Diffraction Extinction” (HRLIRDE). This method is designed around a series of steps involving Fourier transformation, extinction position identification, the construction of extinction diffraction wave functions, filtering, lattice image reconstruction, and real image comparison analysis. Additionally, this study provides theoretical derivations for this method based on phase contrast dynamics [8,16] and high-resolution imaging principles, establishing its theoretical foundation.

Finally, the proposed method was applied to analyze edge and screw dislocations in HRTEM images of electro-deposited copper thin films. The results indicate that this method can effectively identify and determine the Burgers vectors of edge dislocations and screw dislocations. Its main advantage lies in the fact that it only requires high-resolution transmission electron microscopy (HRTEM) images for the analysis of edge and screw dislocations with Burgers vectors $\langle 110\rangle$ and $\langle 112\rangle$. This reduces the complexity and cost of the research. Additionally, this method can also be applied to analyze high-resolution images with reduced clarity, where the dislocation positions are difficult to directly determine using conventional analysis techniques.

## 2. Method

### 2.1. Experimental Preparation for High-Resolution Transmission Electron Microscopy Observation

In this study, Cu films were prepared using the electro-deposition method for HRTEM observations. For the electro-deposition process, a high-purity titanium (Ti) foil (purity ≥ 99.99%, Zhongnuo New Material Company, Beijing, China) was used as the cathode. The choice of Ti as the cathode material was motivated by its inertness, which provides excellent corrosion resistance in both acidic and alkaline environments. Additionally, Ti is easily separable from the electro-deposited layer after deposition compared to steel substrates. Prior to electro-deposition, both the anode material (Cu plate) and cathode material (Ti foil, etc.) underwent pre-treatment procedures, including grinding, polishing, and cleaning, to remove oxide layers from the sample surfaces, ensuring smooth surfaces that are suitable for HRTEM observation.

An acid-based copper sulfate electro-deposition solution was employed, consisting primarily of CuSO_4_·5H_2_O (200 g/L, Komeo Chemical Reagent Company, Tianjin, China), concentrated H_2_SO_4_ (98%, Komeo Chemical Reagent Company, Tianjin, China), and trace amounts of additives, all of which were analytical-grade reagents. During electro-deposition, the solution was continuously stirred at a rate of 300 rpm in a temperature-controlled water bath to maintain uniform composition. The anode and cathode were positioned 10 cm apart, and the electro-deposition was conducted at a constant current density of 30 mA/cm^2^ at 25 °C for 1 h to obtain the copper film. Subsequently, the film was cleaned and removed from the Ti substrate for TEM characterization. The equipment used in this study was a transmission electron microscope (Tecanai G2 F20 S-Twin, FEI Company, Eindhoven, The Netherlands).

### 2.2. HRTEM Observation and Analysis

Cu is an FCC metal, and its full dislocation Burgers vector is $a/2\langle 110\rangle$, while partial dislocations have Burgers vectors such as $a/6\langle 112\rangle$ and $a/3\langle 112\rangle$ [12]. To observe the distribution, decomposition, and synthesis of these dislocations using HRTEM, it is advisable to align the incident electron beam along the zone axis $\langle 110\rangle$ [16]. Therefore, before observation, the zone axis $\left[110\right]$ should be adjusted to ensure that it is parallel to the direction of the incident electron beam.

**Figure 2:**
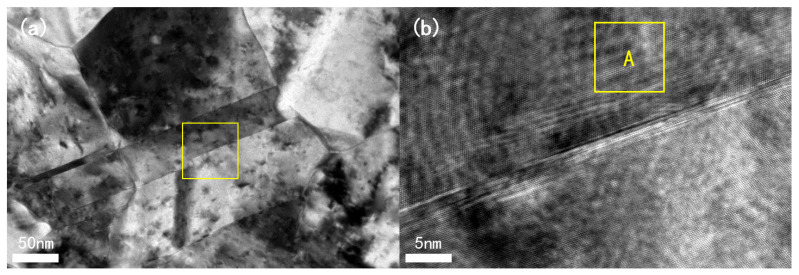
(**a**) Electron diffraction contrast image of twin structure in electroplated copper thin film. An enlarged image of the yellow box is shown in (**b**); (**b**) high-resolution image of the twinned lamella. Region A is the analytical part of the text.

Since this paper primarily focuses on the proposed HRLIRDE method, it is necessary to first locate the dislocations. Due to the potential presence of numerous dislocations within or around the twin boundaries during the formation of twins (the growth mechanism of $a/6\langle 112\rangle$ partial dislocations within twin boundaries [12]) and the relatively common occurrence of Cu metal twinning, this study initially used the electron diffraction contrast imaging (EDCI) technique in TEM to identify the twinning structures in electro-deposited Cu thin films. Subsequently, the analysis mode was switched to high-resolution imaging to obtain HRTEM images. Figure 2 shows the EDCI image of the twinning structure (see Figure 2a) and the corresponding high-resolution image of the twin layers (see Figure 2b).

After obtaining HRTEM images of the twinning in the electro-deposited Cu thin films, we performed the HRLIRDE method. The specific steps are as follows: (1) Perform a Fast Fourier Transform (FFT) on a selected region within the twinning area (e.g., the yellow-boxed region A in Figure 2b) to obtain the Fourier transform spectrum (approximately equivalent to the reciprocal space). (2) Calculate and determine the positions of the Fourier spectrum spots (reciprocal lattice points) corresponding to extinction crystal planes such as {110} and {112} based on the crystallographic angle formula. Virtualize diffraction wave functions corresponding to the extinction spots based on phase contrast dynamics theory, assigning a certain diffraction intensity to these extinction spots. (3) In the Fourier transform spectrum, the diffraction wavefunction corresponding to the extinction point is added to the transmission wavefunction corresponding to the center point, causing mutual interference between the extinction point and the center point. Through the above process, an image of lattice fringes belonging only to the extinction crystal plane is obtained, which helps in the identification of dislocations. This step mainly involves the Fourier inverse transform process. (4) Overlay the obtained fringe images with actual HRTEM images to further confirm the presence and type of dislocations, completing the localization of dislocations. (5) Measure the Burgers vectors of the identified dislocations.

### 2.3. Theoretical Proof of the Analysis Method

Based on the steps described earlier, this study presents the HRLIRDE method, which can be summarized as a process of “transforming high-resolution images into diffraction patterns in reciprocal space through Fourier transformation, selecting diffraction spots corresponding to specific crystal planes, performing an inverse Fourier transform to obtain one-dimensional lattice fringe images corresponding to these crystal planes, and intuitively locating the positions of dislocations”. This process involves existing theories in HRTEM imaging principles, phase contrast dynamics, and diffraction optics. Therefore, this study utilizes the principles of high-resolution transmission electron microscopy imaging, phase contrast dynamics, and diffraction optics to theoretically justify the feasibility of the proposed method. The theoretical proof primarily focuses on whether the interference between the virtualized extinction spot diffraction wave function and the transmission wave function of the central spot in the Fourier transform spectrum can yield periodic lattice fringe images of extinction crystal planes.

**Figure 3:**
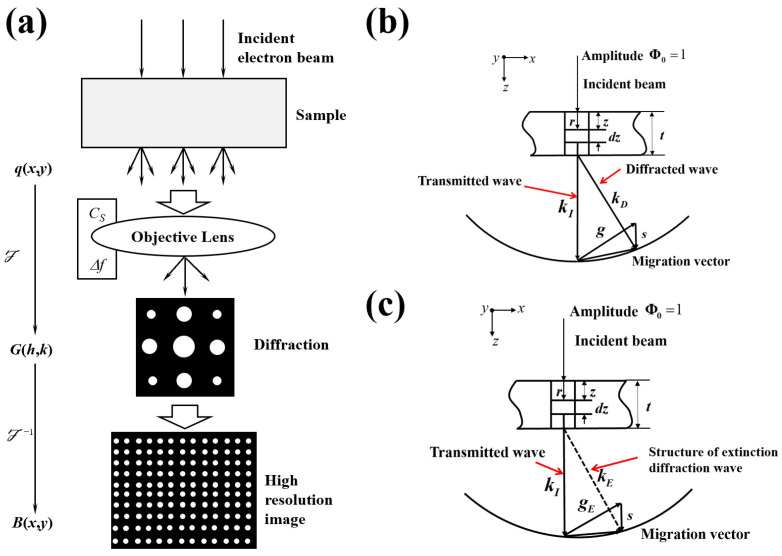
(**a**) Imaging process of HRTEM [18]; (**b**) schematic diagram illustrating the relationship between the transmitted beam and diffracted beam during diffraction under the assumption of the singlecrystal and double-beam approximations; (**c**) schematic diagram illustrating the relationship between the transmitted beam and the extinction-diffracted beam during diffraction extinction under the assumption of the single-crystal and double-beam approximations.

Figure 3a illustrates the schematic process of imaging using HRTEM with phase contrast imaging. In Figure 3b, a schematic representation depicts the relationship between the incident beam and diffracted beam during diffraction under the assumption of a column and double-beam approximation (assuming that there is only one diffracted beam in addition to the transmitted beam) [17]. Figure 3c illustrates the relationship between the incident beam and the extinguished diffracted beam with a structure under the column and double-beam approximation during extinction.

According to Figure 3a, the general imaging process of HRTEM is as follows:

The objective lens performs a Fourier transformation on the surface wave *q*(*x*,*y*) of the sample’s lower surface, resulting in a diffraction wave *G*(*h*,*k*) on the back focal plane:

(1) $$F\left\{q\left(x,y\right)\right\}=G\left(h,k\right)=G\left(g\right)$$

Considering the objective lens aberration (***C_s_***) and defocus (Δf), the diffraction wave *G*(*h*,*k*) is multiplied by a correction factor (phase contrast transfer function exp(i*χ*(*g*)):

(2) $$G\left(h,k\right)=F\left\{q\left(x,y\right)\right\}\cdot \exp\left(i\chi \left(g\right)\right)$$

(3) $$\chi (g)=\chi_{s}+\chi_{d}=\pi (0.5C_{s}\lambda^{3}g^{4}-\Delta f\lambda g^{2})=-\pi \Delta f\lambda g^{2}+\frac{\pi }{2}C_{s}\lambda^{3}g^{4}.$$

where *χ*(*g*) is the phase difference (distortion) function, *χ_s_* and *χ_d_* represent the phase shifts caused by the defocus and aberrations of the HRTEM electromagnetic lens, *λ* is the electron beam wavelength, and *g* is the reciprocal vector length.

On the back focal plane, multiple diffraction orders, including the zeroth-order diffraction (corresponding to the transmitted beam), were selectively filtered using the objective aperture. Coherence was established among these diffraction orders by the objective aperture, and this coherence corresponds to the second Fourier transformation of the acquired $G\left(h,k\right)$. In comparison to the initial Fourier transformation process, this transformation represents a Fourier inverse transformation:

(4) $$B\left(x,y\right)=F^{-1}\left\{G\left(h,k\right)\right\}$$

By performing two Fourier transformations, final lattice and structure images are obtained. At this point, the region of interest in the lattice or structure image can be Fourier transformed; then, select the diffraction spots corresponding to specific crystal planes in the Fourier transform spectrum, filter out noise, and perform an inverse Fourier transform to reconstruct fringe images corresponding to specific crystal planes. Finally, dislocation analysis for specific crystal planes can be conducted based on these fringe images. The inverse Fourier transform process involves coherent implementation using computer programming or existing analysis software and is essentially similar to the aperture coherence in experiments. If the transmitted beam and diffraction spot with reciprocal vector $\vec{g}$ are selected in the Fourier spectrum, coherent analysis is conducted using the transmitted beam and diffraction wave functions, with reciprocal vector $\vec{g}$ corresponding to the selected crystal plane. Therefore, the inverse Fourier transformation process can be specifically written as

(5) $$\psi =\phi_{0}\left(z\right)\exp2\pi i\left({\vec{k}}_{I}\cdot \vec{r}\right)+\phi_{\vec{g}}\left(z\right)\exp2\pi i\left({\vec{k}}_{D}\cdot \vec{r}\right)$$

In the equation, ψ represents the interference wave function, $\phi_{0}\left(z\right)$ denotes the amplitude of the transmitted electron beam wave, $\phi_{\vec{g}}\left(z\right)$ stands for the amplitude of the diffracted electron beam wave, *z* represents the coordinate along the depth direction of the sample, and ${\vec{k}}_{I}$ and ${\vec{k}}_{D}$ are, respectively, the wave vectors of the transmitted and diffracted electron beams. $\exp2\pi i\left({\vec{k}}_{I}\cdot \vec{r}\right)$ can be considered the phase factor of the transmitted wave, and $\exp2\pi i\left({\vec{k}}_{D}\cdot \vec{r}\right)$ can be considered the phase factor of the diffracted wave. Clearly, Equation (5) satisfies the definition of Fourier (inverse) transformation.

Suppose the reciprocal vector is $\vec{g}$, and the deviation vector from the reciprocal point is $\vec{s}$, as shown in Figure 3b. Then, according to the definition of the deviation vector [17], we can obtain the following:

(6) $${\vec{k}}_{D}={\vec{k}}_{I}+\vec{g}+\vec{s}$$

Introducing the double-beam approximation [17], based on phase contrast dynamics [17], it can be stated that

(7) $$\phi_{\vec{g}}=i\exp\left(\pi isz\right)\sin\beta \sin\left(\pi \Delta Kz\right)$$

where *z* is the depth into the crystal from the upper surface; β=arccotω, $\omega =s\xi_{\vec{g}}$, and $\xi_{\vec{g}}$ are extinction distances; $\Delta K=\frac{\sqrt{1+\omega^{2}}}{\xi_{\vec{g}}}$.

For a sample with thickness ***t***, in Equation (7), z=t. Additionally, based on trigonometric relationships, it can be obtained that

(8) $$\sin\beta =\sin\left(\mathrm{arc}\cot\omega \right)=\frac{1}{\sqrt{1+\omega^{2}}}=\frac{1}{\sqrt{1+s^{2}\xi_{\vec{g}}^{2}}}=\frac{1}{\xi_{\vec{g}}\sqrt{\xi_{\vec{g}}^{-2}+s^{2}}}=\frac{1}{\xi_{\vec{g}}s_{eff}}$$

where $s_{eff}$ is the effective deviation vector:

(9) $$s_{eff}=\sqrt{s^{2}+\xi_{\vec{g}}^{-2}}$$

The effective deviation vector defined by Equation (9) is represented by $\Delta K=s_{eff}$, and according to Euler’s formula, we have that

(10) $$\sin\left(\pi \Delta Kz\right)=\sin\left(\pi s_{eff}t\right)$$

(11) $$\begin{array}{c} \phi_{\vec{g}}=\phi_{\vec{g}}=i\exp\left(\pi isz\right)\sin\beta \sin\left(\pi \Delta Kz\right)=\frac{1}{\xi_{\vec{g}}s_{eff}}\sin\left(\pi s_{eff}t\right)\left(-sin\left(\pi st\right)+i\cos\left(\pi st\right)\right)\\ =\left(\frac{\pi t}{\xi_{\vec{g}}}\cdot \frac{\sin\left(\pi s_{eff}t\right)}{\pi s_{eff}t}\right)\exp i\left(\frac{\pi }{2}+\pi st\right) \end{array}$$

Substituting Equations (6), (7), and (11) into Equation (5) and using complex variable conjugate transformations as well as trigonometric functions, it can be derived that

(12) $$\psi =\exp2\pi i\left(k_{I}\cdot \vec{r}\right)\left[\phi_{0}\left(z\right)+\left(\frac{\pi t}{\xi_{\vec{g}}}\frac{\sin\left(\pi s_{eff}t\right)}{\pi s_{eff}t}\right)\exp i\left(\left(\vec{g}+\vec{s}\right)\cdot \vec{r}+\frac{\pi }{2}+\pi st\right)\right]$$

When approximating that $\vec{g}+\vec{s}$ is parallel to the *x*-axis, the final result yields the intensity of the interference wave as

(13) $$I={\left|\psi \right|}^{2}=\psi^{*}\cdot \psi =\phi_{0}^{2}\left(z\right)+{\left(\frac{\pi t}{\xi_{\vec{g}}}\frac{\sin\left(\pi s_{eff}t\right)}{\pi s_{eff}t}\right)}^{2}-2\phi_{0}\left(z\right)\left(\frac{\pi t}{\xi_{\vec{g}}}\frac{\sin\left(\pi s_{eff}t\right)}{\pi s_{eff}t}\right)\sin\left(2\pi \left|\vec{g}+\vec{s}\right|x+\pi st\right)$$

From Equation (13), it can be observed that the image obtained by interfering the transmitted beam with the diffracted beam with a reciprocal vector of $\vec{g}$ is an intensity distribution with sinusoidal oscillations, and its period is

(14) $$\frac{2\pi }{2\pi \cdot \left|\vec{g}+\vec{s}\right|}=1/\left|\vec{g}+\vec{s}\right|$$

Since $\left|\vec{s}\right|$ is much smaller than $\left|\vec{g}\right|$ by up to three orders of magnitude [19], it can be assumed that the period of the interference image is approximately $1/\left|\vec{g}\right|$. According to the properties of the reciprocal lattice, the length of the reciprocal vector is equal to the reciprocal of the lattice spacing d of the corresponding crystallographic plane, which implies that $1/\left|\vec{g}\right|=d$. Therefore, it can be inferred that when the transmitted beam is interfered with the diffracted beam that has a reciprocal vector of $\vec{g}$, the period of the resulting interference image can be approximately considered to be the spacing of the crystallographic plane corresponding to the selected reciprocal vector $\vec{g}$, and the interference image represents the fringes corresponding to the reciprocal vector $\vec{g}$ that is associated with the crystallographic plane.

Based on the above derivation, if the position of the extinction spot is calculated in the diffraction pattern at this point, it corresponds to ${\vec{g}}_{E}$. At this stage, the diffraction beam’s wave function corresponding to the extinction spot can be constructed according to Equation (11):

(15) $$\phi_{{\vec{g}}_{E}}=B\exp i\left(\frac{\pi }{2}+\pi st\right)\exp2\pi i({\vec{k}}_{E}\cdot \vec{r})$$

In Equation (15), ${\vec{k}}_{E}$ is the wave vector of the constructed diffraction wave, and its relationship with the wave vector of the incident wave ${\vec{k}}_{I}$ is shown in Figure 3c and can be written as

(16) $${\vec{k}}_{E}={\vec{k}}_{I}+{\vec{g}}_{E}+\vec{s}$$

After derivation, it can be determined that the intensity of the interference between the extinction spots and the transmission spots is given by

(17) $$I=\phi_{0}^{2}\left(z\right)+B^{2}-2\phi_{0}\left(z\right)B\sin\left(2\pi \left|{\vec{g}}_{E}+\vec{s}\right|x+\pi st\right)$$

From Equation (17), it can be deduced that the obtained interference image is also an intensity distribution with sinusoidal oscillations, and its period is $1/\left|{\vec{g}}_{E}+\vec{s}\right|$. Clearly, this period spacing is approximately equal to the lattice spacing of the extinction crystallographic plane. Therefore, the obtained image can be regarded as the fringe image of the extinction crystallographic plane. At this point, the obtained image contains only the fringe patterns of the extinction crystallographic plane, which is suitable for analyzing full dislocations and partial dislocations that are involved in the extinction crystallographic plane.

In summary, the proposed HRLIRDE is theoretically valid.

### 2.4. Extinction Speckle Reconstruction and Dislocation Verification Methods

**Figure 4:**
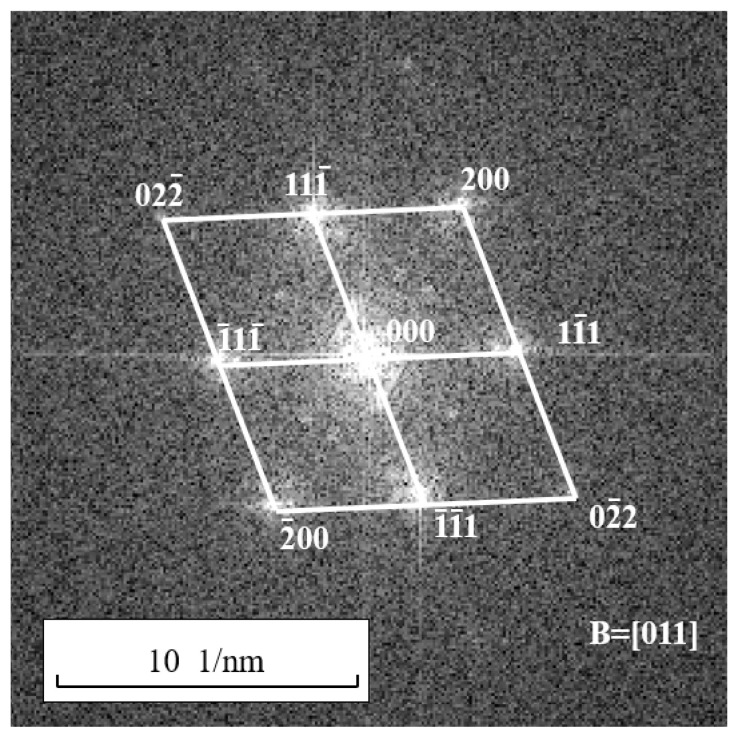
The Fourier transform spectrum of region A in Figure 2b.

Region A in the HRTEM image shown in Figure 2 was subjected to a Fast Fourier Transform (FFT) to obtain the corresponding Fourier transform spectrum, as shown in Figure 4. This Fourier transformation is equivalent to transforming Equation (4) into $G\left(h,k\right)$ in Equation (2). The resulting Fourier transform spectrum closely resembles the reciprocal lattice. After calibrating the Fourier transform spectrum, it can be observed that there are no spots corresponding to {112} and {110} crystallographic planes. Therefore, it is not possible to directly observe the periodic information of the {112} and {110} crystallographic planes, as well as the corresponding dislocations such as full dislocations like $a/2\langle 110\rangle$ and partial dislocations like $a/6\langle 112\rangle$ and $a/3\langle 112\rangle$, through subsequent filtering and inverse Fourier transformation in the HRTEM image.

At this point, the HRLIRDE proposed in this study can be employed to address this issue.

It should be noted that, according to the HRLIRDE method proposed in this study, after locating the position of the extinction spots, it is necessary to virtually construct diffraction wavefunctions for the extinction spots based on the theory of diffraction contrast and assign a certain diffraction intensity to these extinction spots.

Taking the $\left(21\overset{-}{1}\right)$ plane in the FCC $\left\{211\right\}$ crystal family as an example, according to the formula [12] for interplanar spacing in cubic crystal systems,

(18) $$d=a/\sqrt{h^{2}+k^{2}+l^{2}}$$

where *d* represents the interplanar spacing; *a* is the lattice constant of the Cu crystal, which is 0.36 nm [20]; and *h*, *k*, and *l* are the Miller indices for the plane, and in this case, they are taken as 2, 1, and −1, respectively. Using these values, the interplanar spacing for the $\left(21\overset{-}{1}\right)$ plane can be calculated as 0.15 nm. According to the formula [12] for the angular relationship between crystal planes in a cubic crystal system,

(19) $$\cos\varphi =(h_{1}h_{2}+k_{1}k_{2}+l_{1}l_{2})/\sqrt{\left({h_{1}}^{2}+{k_{1}}^{2}+{l_{1}}^{2})({h_{2}}^{2}+{k_{2}}^{2}+{l_{2}}^{2}\right)}$$

**Figure 5:**
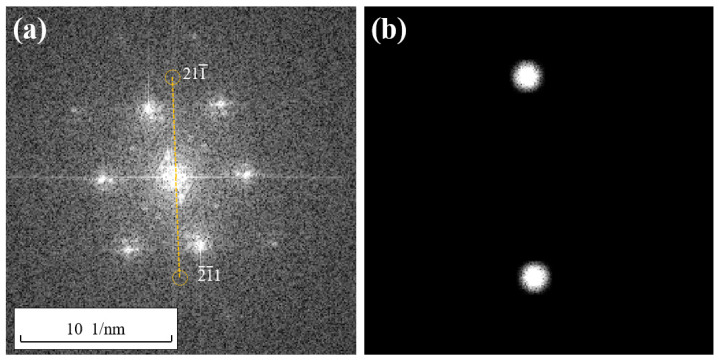
(**a**) The position of $\left(21\overset{-}{1}\right)$ plane dimming spot in a reciprocal lattice (extinction spots symmetrical to the center and their connecting lines are identified in the figure); (**b**) the filtered image.

The angle between the $\left(21\overset{-}{1}\right)$ plane and the $\left(11\overset{-}{1}\right)$ plane is calculated to be 19°, and the angle between the $\left(21\overset{-}{1}\right)$ plane and the (200) plane is calculated to be 35°. Based on this information, the positions of the extinction spots corresponding to the $\left(21\overset{-}{1}\right)$ plane can be determined in the reciprocal lattice, along with the positions of another extinction spot $\left(\overset{-}{2}\overset{-}{1}1\right)$ that is symmetrically opposite to it through the center of the Fourier transform spectrum, as shown in Figure 5a. Wave functions are constructed for these two points, and filtering is applied. The results are shown in Figure 5b, where the two symmetric spots in Figure 5b represent the shapes of the $\left(\overset{-}{2}\overset{-}{1}1\right)$ plane extinction spots after filtering.

**Figure 6:**
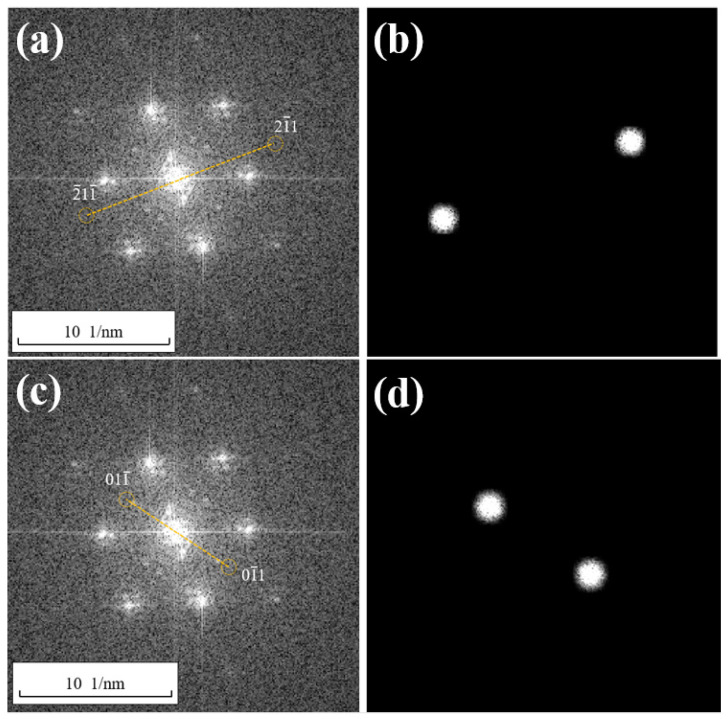
The position of $\left(2\overset{-}{1}1\right)$ (**a**) and $\left(01\overset{-}{1}\right)$ (**c**) (extinction spots symmetrical to the center and their connecting lines are identified in the figure) dimming spot in a reciprocal lattice; the filtered image of $\left(2\overset{-}{1}1\right)$ (**b**) and $\left(01\overset{-}{1}\right)$ (**d**).

Consider another $\left(2\overset{-}{1}1\right)$ crystal plane within the $\left\{211\right\}$ crystallographic family. Similar to the calculation process described earlier, the angle between the planes $\left(2\overset{-}{1}1\right)$ and $\left(11\overset{-}{1}\right)$ can be calculated using Equations (18) and (19) to be 90°. When the angle between the planes $\left(2\overset{-}{1}1\right)$ and (200) is 35°, and the interplanar spacing of the $\left(2\overset{-}{1}1\right)$ crystal planes is 0.15 nm, the position is determined, and filtering is applied. The resulting $\left(2\overset{-}{1}1\right)$ spots’ positions in the Fourier transform spectrum and the construction of the wave function, as well as the filtered spot morphology, are shown in Figure 6a,b. The positions and filtered spot morphology of other crystal plane spots within the $\left\{211\right\}$ crystallographic family in the Fourier transform spectrum are not further elaborated.

Finding spots on $\left(01\overset{-}{1}\right)$ crystal planes that are extinguished due to odd–even mixing is relatively simple. They can be directly derived from the unextinguished $\left(02\overset{-}{2}\right)$ spots in the Fourier transform spectrum, located at the midpoint between the $\left(02\overset{-}{2}\right)$ and transmitted spots, as shown in Figure 6c. After filtering the extinguished $\left(01\overset{-}{1}\right)$ spots, their spot morphology is as shown in Figure 6d.

Finally, after observing the dislocations using the method proposed in this study, the observed dislocation Burgers vector lengths can be compared with the theoretically calculated Burgers vector lengths, which can be used to verify the correctness of the dislocation analysis. In FCC crystals, their length can be calculated using formula [12]:

(20) $$\left|b\right|=a/n\cdot \sqrt{u^{2}+v^{2}+w^{2}}$$

In Equation (20), *a*/*n* represents a fractional multiple of the lattice constant; *u*, *v*, and *w* are the Miller indices of the crystallographic plane.

## 3. Result Analysis and Discussion

### 3.1. Observation and Analysis of Edge Dislocations

**Figure 7:**
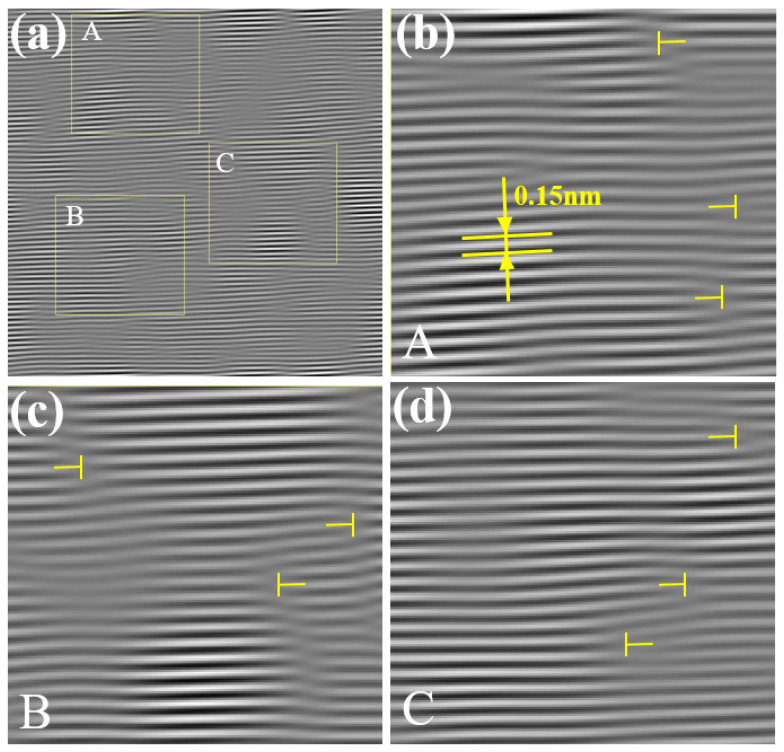
A wide distribution of dislocations (marked with a yellow “T”) and plane spacing can be seen in the inverse Fourier transform of the filtered dimming spots on the $\left(21\overset{-}{1}\right)$ plane; (**a**) is the original image, (**b**–**d**) are magnified images of regions A, B, and C, respectively, identified in (**a**).

After the construction of wave functions and filtering, the $\left(21\overset{-}{1}\right)$ extinction spot in Figure 5 was interfered with the central spot in the Fourier transform spectrum using Equations (5)–(17). This process is also known as an Inverse Fast Fourier Transform (IFFT) process. The resulting fringe pattern is shown in Figure 7, and according to Equation (17), it can be considered the lattice fringe pattern of the $\left(21\overset{-}{1}\right)$ crystallographic plane. Moreover, through the above approach, Figure 7 primarily displays the lattice fringe pattern of the $\left(21\overset{-}{1}\right)$ crystallographic plane, containing the periodic information that is enhanced by the crystallographic plane. This is more conducive to locating positions with incomplete periodicity in the lattice fringe pattern through analysis, and thereby determining the distribution of edge dislocations that are generated by the $\left(21\overset{-}{1}\right)$ crystallographic plane.

The lattice fringe pattern shown in Figure 7 was subjected to fringe spacing measurements, and the measured spacing was determined to be 0.153 nm, which is essentially the same as the previously calculated spacing of 0.15 nm for the $\left(21\overset{-}{1}\right)$ crystallographic plane. This confirms that Figure 7 represents the lattice fringe pattern of the $\left(21\overset{-}{1}\right)$ crystallographic plane. From Figure 7, it can be visually observed that there are numerous edge dislocations within the entire region, which may be related to the selected area being near a twin boundary [12,13].

**Figure 8:**
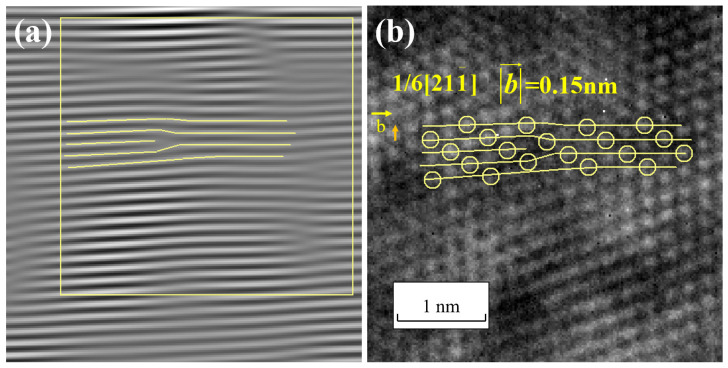
Morphology of $1/6[21\overset{-}{1}]$ dislocation in one-dimensional lattice fringe image (**a**) and two-dimensional lattice image (**b**).

To further analyze the dislocation morphology in region A of Figure 7, which contains lattice distortions at the center of the image, the various crystal planes that make up a dislocation in region A are marked with lines, as shown in Figure 8a. Then, these lines that are used for marking are placed in the corresponding positions within the two-dimensional lattice image, as shown in Figure 8b. Since the one-dimensional fringe pattern essentially retains the information of crystal planes in one direction, the distribution and position information of the atoms remain unchanged. Therefore, as long as the relative positions of the marking lines in the field of view remain unchanged, it is possible to identify the corresponding atoms in Figure 8b based on the lines and depict the specific morphology of the dislocation. The total amount of lattice distortion caused by this dislocation is the magnitude of the Burgers vector of the dislocation, which can be measured to be 0.15 nm in size in the direction of vector $\left[21\overset{-}{1}\right]$.

In FCC crystals, the Burgers vector of a Shockley partial dislocation is denoted as $a/6\langle 112\rangle$ [12], and its length can be calculated using Formula (20). From this, the magnitude of the Burgers vector for the Shockley partial dislocation a/6<112> can be calculated as $\sqrt{6}a/6$. Using the previously provided lattice constant *a* for Cu, which is 0.36 nm, the calculated magnitude of the Burgers vector $\left|b\right|$ for the Shockley partial dislocation a/6<112> in Cu is 0.15 nm. This result is in excellent agreement with the magnitude of the Burgers vector that was measured from the Burgers circuit in Figure 8b. Thus, it can be concluded that a Shockley partial dislocation has been identified in region A of Figure 7b, and its Burgers vector has been determined to be $1/6[21\overset{-}{1}]$.

**Figure 9:**
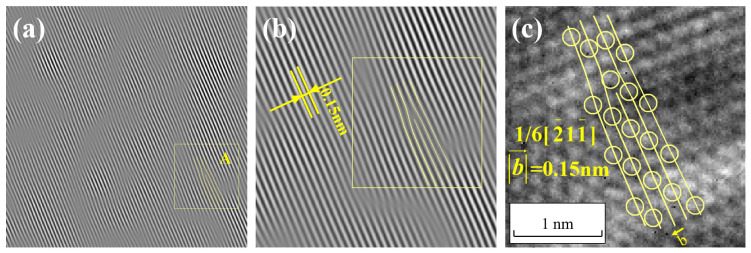
Morphology of $1/6[\overset{-}{2}1\overset{-}{1}]$ dislocation in one-dimensional lattice fringe image (**a**,**b**) and two-dimensional lattice image (**c**).

After performing the interference between the $\left(2\overset{-}{1}1\right)$ extinction spots that were obtained from Figure 6b through the construction of the wave function and filtering and the central spot in the Fourier transform spectrum using Equations (5)–(17), the lattice fringe pattern shown in Figure 9a is obtained. Region A from Figure 9a is selected and enlarged to produce Figure 9b, which is then compared to the experimental HRTEM lattice image, as shown in Figure 9c. From the lattice fringe pattern, the interplanar spacing is measured to be 0.15 nm, which is consistent with the calculated interplanar spacing of the $\left(2\overset{-}{1}1\right)$ crystal plane. This confirms that the lattice fringe pattern belongs to the crystallographic family of the $\left(2\overset{-}{1}1\right)$ crystal plane.

By selecting the boxed area for the dislocation analysis and using the previously described dislocation analysis method, the morphology of the dislocation can be observed in the two-dimensional lattice image, and the magnitude of the Burgers vector is measured to be 0.15 nm in the direction of vector $\left[\overset{-}{2}1\overset{-}{1}\right]$. This Burgers vector magnitude matches the Burgers vector magnitude of the Shockley partial dislocation a/6<112> in Cu, and it can be concluded that a Shockley partial dislocation with a Burgers vector $1/6[\overset{-}{2}1\overset{-}{1}]$ has been found in Figure 9c.

**Figure 10:**
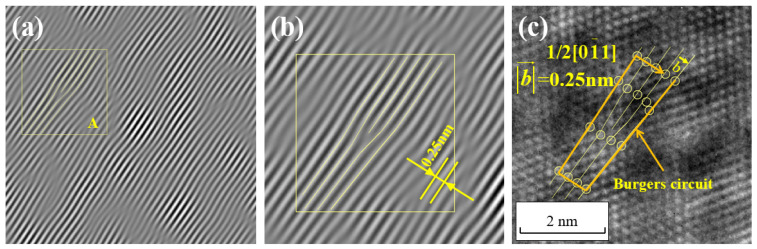
Morphology of $1/2[0\overset{-}{1}1]$ dislocation in one-dimensional lattice fringe image (**a**,**b**) and two-dimensional lattice image (**c**).

After performing the interference between the $\left(0\overset{-}{1}1\right)$ extinction spots that were obtained from Figure 6d through the construction of the wave function and filtering and the central spot in the Fourier transform spectrum using Equations (5)–(17), the lattice fringe pattern shown in Figure 10a is obtained. Region A from Figure 10a is selected and enlarged, and then compared to the experimental HRTEM lattice image, as shown in Figure 10c. The measured interplanar spacing is found to be 0.25 nm, which matches the calculated interplanar spacing of the $\left(0\overset{-}{1}1\right)$ crystal plane. This confirms that the lattice fringe pattern in Figure 10 corresponds to the lattice fringe pattern of the $\left(0\overset{-}{1}1\right)$ crystal plane.

For the boxed area, a dislocation analysis is conducted. The Burgers vector magnitude of the dislocation is measured to be 0.25 nm in the direction of vector $\left[0\overset{-}{1}1\right]$. This Burgers vector magnitude matches the magnitude of the Burgers vector for a full dislocation a/2<110> in Cu. Therefore, it can be concluded that a full dislocation with a Burgers vector $1/2[0\overset{-}{1}1]$ has been found in Figure 10c.

In Figure 8b and Figure 9c, it can be observed that the atoms constituting the $\left(21\overset{-}{1}\right)$ and $\left(2\overset{-}{1}1\right)$ exhibit a discontinuous distribution, making it challenging to directly discern their distribution characteristics. It is difficult to identify, assess, and measure the Burgers vector directly from the atomic arrangement. Clearly, the HRLIRDE method proposed in this study can effectively address this issue and offers unique advantages.

### 3.2. Observation and Analysis of Screw Dislocations

Screw dislocations are a crucial type of defect in metals [21,22,23,24,25,26]. Although their presence can be determined through conventional electron diffraction two-beam methods [19], observing screw dislocations at the atomic scale has remained challenging. Yang et al. achieved atomic-scale imaging of screw dislocations in a scanning transmission electron microscope using a method involving aberration-corrected electron optical slicing [27]. This method involved controlling the depth of each scan to obtain the positional information of several atomic layers and reconstructing the complete structural image. However, this approach is relatively complex and places high requirements on the equipment. In this paper, we present a relatively simple and universally applicable high-resolution transmission electron microscopy method for observing screw dislocations, based on the HRLIRDE method.

**Figure 11:**
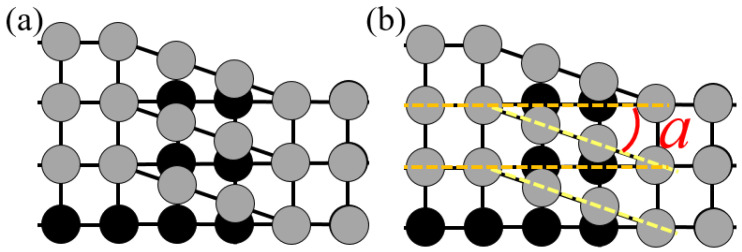
Periodic structure (**a**) of screw dislocation and schematic diagram (**b**).

Screw dislocations [28] essentially involve the upper-layer atoms sliding parallel to the dislocation line relative to the lower-layer atoms. This results in lattice distortion in the upper layer, as shown in Figure 11a. From the schematic diagram, it can be seen that while the overall lattice undergoes twisting and distortion, local periodic arrangements still exist, specifically, periodic arrangements on the left side of the dislocation line and distorted lattice on the right side of the dislocation line. The angle between the lattices on either side of the periodic arrangement is denoted as *a*, as depicted in Figure 11b. Consequently, for upper-layer atoms, the spacing between the regular lattice and the distorted lattice planes can be approximated as identical, with an angle *α* existing in that direction. In reciprocal space, this is manifested as two reciprocal lattice points that are equidistant from the reciprocal origin and with an *α* angle in the connecting line.

**Figure 12:**
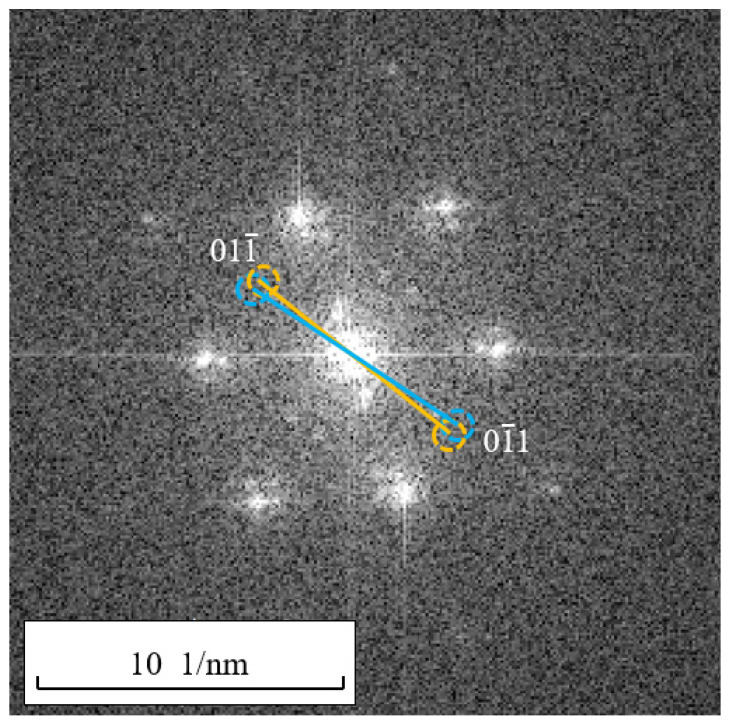
Filtering of two pairs of reciprocal points with similar angles.

Common in FCC metals are $a/2\langle 110\rangle$ screw dislocations [18,28]. The $\left\{110\right\}$ crystallographic plane, perpendicular to the $\langle 110\rangle$ direction, is the extinction crystallographic plane. Therefore, we still employ the approach outlined in Section 3.2 to identify the $01\bar{1}$ extinction spots in the Fourier transform spectrum and their symmetric counterparts $0\bar{1}1$. Subsequently, we rotate a reciprocal lattice vector connecting the $01\bar{1}$ extinction spots, the center of the Fourier transform spectrum, and the $0\bar{1}1$ spots by an angle, such as 5°, to obtain two new symmetric spots, as illustrated in Figure 12. Evidently, the reciprocal vector lengths of these two new spots remain unchanged, and they can be regarded as the extinction spots corresponding to the $01\bar{1}$ crystal planes rotated by an angle. Coherently combining these two sets of extinction spots, which have an angle of 5°, in a reciprocal space with the central spot in the Fourier spectrum generates periodic fringe patterns is an attempt to identify whether the patterns resemble those shown in Figure 11. Since the extent of lattice distortion caused by screw dislocations cannot be predicted, multiple attempts may be required if coherent combinations of the two sets of spots do not yield periodic structures resembling those shown in Figure 11. In this case, a 5° rotation was chosen, which produced the expected results.

**Figure 13:**
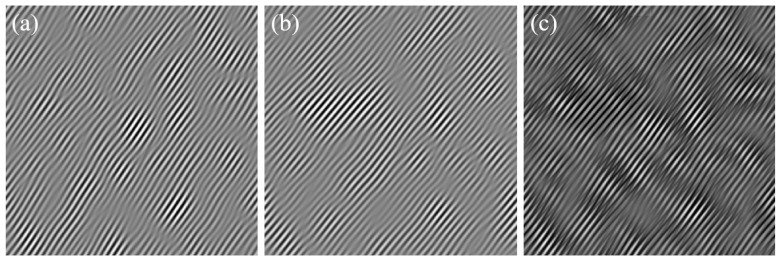
Lattice fringe images (**a**,**b**) and superimposed lattice fringe image (**c**).

**Figure 14:**
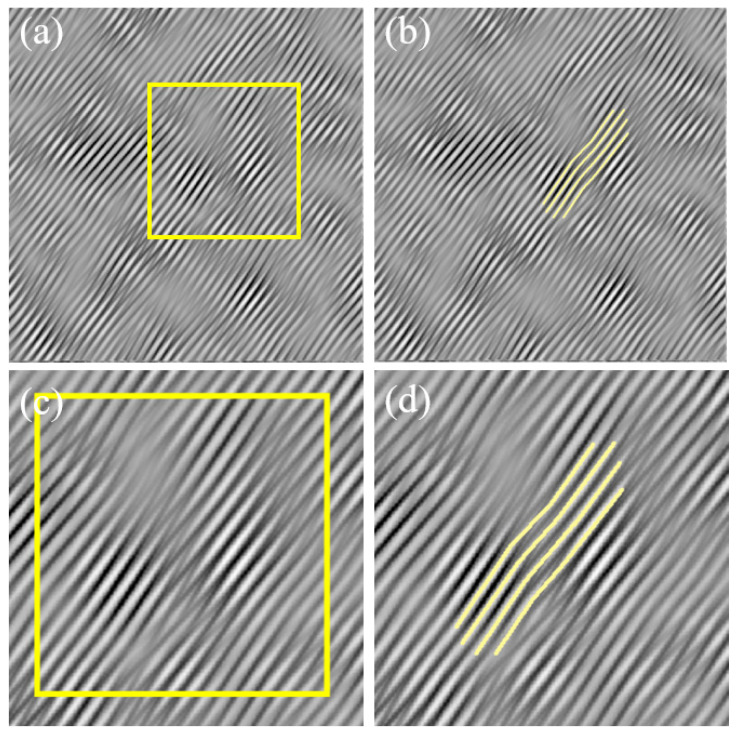
Lattice fringe superposition image (**a**,**c**) and part of it with screw dislocation morphology (**b**,**d**).

After constructing the diffraction wave functions and performing filtering, the periodic fringe patterns that were obtained by interfering the two pairs of $01\bar{1}$ extinction spots with the center spot of the Fourier transform spectrum, as shown in Equations (5)–(17), are depicted separately in Figure 13a,b. To observe the periodic relationship more clearly between the two images, the lattice fringe patterns from both figures are superimposed, as shown in Figure 13c. In the superimposed image, the typical lattice arrangement of upper-layer atoms in a screw dislocation is discernible. It is marked and magnified, as depicted in Figure 14.

**Figure 15:**
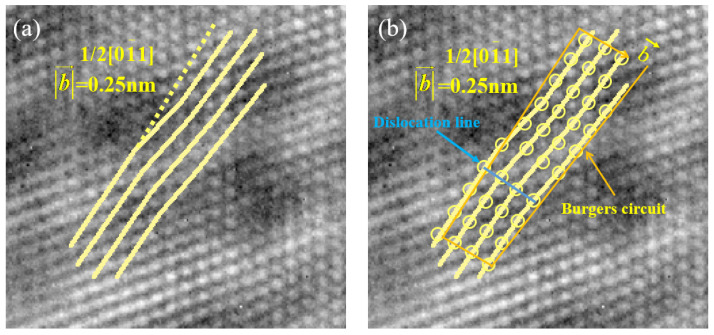
(**a**) A screw dislocation in two-dimensional lattice images; (**b**) The Burgers circuit of the screw dislocation.

Furthermore, the distorted crystal planes are marked at the corresponding positions in the lattice image, as depicted in Figure 15. The total amount of lattice distortion can be measured from the image, yielding a value of 0.25 nm. This indicates that the Burgers vector magnitude for this screw dislocation is 0.25 nm, which is identical to the size of a/2<110> dislocations in Cu. The direction of the Burgers vector is $\left[01\overset{-}{1}\right]$, confirming the identification of a screw dislocation with a Burgers vector of $1/2[01\overset{-}{1}]$.

Obviously, for this method of finding and measuring screw dislocation, it is necessary to use a computer to carry out continuous calculation and reconstruction with different angles and compare with the real HRTEM structural image. It is worth mentioning that since the method of analyzing screw dislocations involves some repetitive work, the workload may be greatly enhanced by the experimental purpose and experimental conditions, and perhaps the efficiency of the analysis can be improved by combining this method with some AI algorithms, just as AI algorithms are used in other fields [29,30].

## 4. Conclusions

This paper presents a method for high-resolution lattice image reconstruction and dislocation analysis based on diffraction extinction. The approach primarily involves locating extinction spots in the Fourier transform spectrum (reciprocal space) and virtually constructing the corresponding diffraction wave functions. Subsequently, the filtered diffraction and transmission waves are coherently combined to reconstruct the lattice image of the extinction planes. This enables the observation of crystal facets that exhibit electron diffraction extinction effects and an atomic jump arrangement during high-resolution transmission electron microscopy (HRTEM) characterization. Simultaneously, since this method can be used to locate dislocations based on lattice fringe images, it can also facilitate dislocation analysis in high-resolution images with suboptimal quality.

(1) Based on the principles of high-resolution transmission electron microscopy, diffraction dynamics, and diffraction optics, it can be deduced that after constructing diffraction wave functions for extinction spots, the overlaid interference of the diffraction wave functions and transmission wave functions yields the periodic lattice image of the extinction crystal plane, with a period that is approximately equal to the crystal plane spacing. This demonstrates the theoretical feasibility of the method that is proposed in this paper.
(2) Using the diffraction extinction-based high-resolution lattice image reconstruction and metal dislocation analysis method, this study characterized edge dislocations within Cu films that were prepared by electrodeposition. It successfully observed dislocations like $1/6[21\overset{-}{1}]$, $1/6[\overset{-}{2}1\overset{-}{1}]$, and $1/2[0\overset{-}{1}1]$, which are challenging to observe in conventional high-resolution transmission electron microscopy characterization.
(3) Building upon the above method, this paper also characterized screw dislocations that are difficult to observe in high-resolution transmission electron microscopy. Through repeated attempts to superimpose lattice fringe images that were obtained by reconstructing extinction spots with a certain angular deviation, a screw dislocation with a Burgers vector of $1/2[01\overset{-}{1}]$ was successfully observed.
(4) The method described in this paper can theoretically be widely used in transmission electron microscopy-based dislocation analysis, but it may be necessary to find appropriate substitutions for Formulas (18)–(20) and to substitute suitable parameters in the individual formulas when the crystals are not FCC-structured.

The research method presented in this paper allows for the analysis and study of dislocations with Burgers vectors of $\langle 110\rangle$ and $\langle 112\rangle$ based solely on high-resolution images captured by HRTEM. This reduces the complexity and cost of research, expanding the application of general high-resolution transmission electron microscopy in microscale observations.

## Data Availability

Data are contained within the article.
